# A De Novo Transcriptome Assembly of *Ceratopteris richardii* Provides Insights into the Evolutionary Dynamics of Complex Gene Families in Land Plants

**DOI:** 10.1093/gbe/evab042

**Published:** 2021-03-03

**Authors:** Yuan Geng, Chao Cai, Scott A.M McAdam, Jo Ann Banks, Jennifer H Wisecaver, Yun Zhou

**Affiliations:** 1 Department of Botany and Plant Pathology, Purdue University, West Lafayette, Indiana, USA; 2 Purdue Center for Plant Biology, Purdue University, West Lafayette, Indiana, USA; 3 Purdue University Libraries and School of Information Studies, Purdue University, West Lafayette, Indiana, USA; 4 Department of Biochemistry, Purdue University, West Lafayette, Indiana, USA

**Keywords:** Ceratopteris transcriptome, ferns, evolutionary dynamics, complex gene families, molecular evolution, GRAS domain proteins

## Abstract

As the closest extant sister group to seed plants, ferns are an important reference point to study the origin and evolution of plant genes and traits. One bottleneck to the use of ferns in phylogenetic and genetic studies is the fact that genome-level sequence information of this group is limited, due to the extreme genome sizes of most ferns. *Ceratopteris richardii* (hereafter Ceratopteris) has been widely used as a model system for ferns. In this study, we generated a transcriptome of Ceratopteris, through the de novo assembly of the RNA-seq data from 17 sequencing libraries that are derived from two sexual types of gametophytes and five different sporophyte tissues. The Ceratopteris transcriptome, together with 38 genomes and transcriptomes from other species across the Viridiplantae, were used to uncover the evolutionary dynamics of orthogroups (predicted gene families using OrthoFinder) within the euphyllophytes and identify proteins associated with the major shifts in plant morphology and physiology that occurred in the last common ancestors of euphyllophytes, ferns, and seed plants. Furthermore, this resource was used to identify and classify the GRAS domain transcriptional regulators of many developmental processes in plants. Through the phylogenetic analysis within each of the 15 GRAS orthogroups, we uncovered which GRAS family members are conserved or have diversified in ferns and seed plants. Taken together, the transcriptome database and analyses reported here provide an important platform for exploring the evolution of gene families in land plants and for studying gene function in seed-free vascular plants.

**Significance**

Ferns are an important reference point to study the origin and evolution of plant genes and traits. *Ceratopteris richardii* (Ceratopteris) has been widely used as the model system for ferns. Although a partial assembly representing 38% of the nuclear genome of Ceratopteris was recently published, the large size (11.25 Gb) and high complexity of the Ceratopteris genome has hindered the comprehensive investigation into the protein coding repertoire of this model species. In this study, we report a transcriptome of Ceratopteris, through the de novo assembly of RNA-seq libraries derived from two sexual types of gametophytes and five different sporophyte tissues. We leverage the genomes and transcriptomes of 38 additional plant species across the Viridiplantae to assign Ceratopteris predicted proteins to 17,489 orthologous families. Phylogenomic analysis of these gene families shows the dynamic gain, loss and amplification of different orthogroups in the last common ancestor of the major plant lineages, including in the sister lineages of ferns and seed plants. Using this resource, we also characterize the evolutionary history of GRAS domain proteins and uncover both conserved and lineage-specific GRAS subfamilies in land plants.

## Introduction

Ferns (Monilophyta) are the second most diverse group of vascular plants, with >10,000 species (PPG I 2016). Ferns are also a unique plant lineage, possessing both ancestral traits (e.g., spores and independent gametophytes) as well as many derived traits (e.g., vascular systems and true leaves) that they share with their larger and iconic sister lineage, the seed plants (Banks 1999; Plackett et al. 2015). Despite the fact that ferns represent a critical branch of the land plant tree of life, they are often absent from investigations into the origin and evolution of plant traits (Plackett et al. 2015; Rensing 2017). The reason for this omission is practical; ferns have extremely large genomes that average over 10 Gb in length (Sessa and Der 2016). Genome assemblies are available for only two fern species, *Azolla filiculoides* and *Salvinia cucullata*, both of which are heterosporous and have small genomes (0.75 Gb and 0.25 Gb, respectively) that have been secondarily reduced (Li et al. 2018). This leaves the homosporous ferns and their enormous genomes with little to no representation in genome-level analyses of plant evolution.

The homosporous fern Ceratopteris is a model system for studying many aspects of fern biology and especially gametophyte biology, including sex determination (Hickok et al. 1987; Banks et al. 1993; Banks 1994, 1997, 1999; Atallah and Banks 2015), physiology (McAdam et al. 2016), spore germination (Chatterjee and Roux 2000; Salmi et al. 2005), gametophyte meristem development (Banks 1999; Plackett et al. 2018), and photomorphogenesis (Cooke et al. 1995). Although a partial assembly representing 38% of the nuclear genome of Ceratopteris was recently published (Marchant et al. 2019), the large size (11.25 Gb) and high complexity of the Ceratopteris genome has hindered comprehensive investigation into the gene repertoire of this model fern. The 1,000 plants (oneKP) initiative published de novo transcriptome assemblies for Ceratopteris and several other diverse fern species (Leebens-Mack et al. 2019). However, the oneKP transcriptomes are mainly derived from sporophyte leaf tissue, which is sufficient for taxonomic studies of conserved gene families but will miss critical genes that are not expressed in leaves (e.g., gametophyte-specific genes).

Here, we report a Ceratopteris transcriptome database, which we de novo assembled using 17 RNAseq libraries from two sexual types of gametophytes (Atallah et al. 2018) and five different sporophyte tissues. This assembly captures protein sequences from 44,668 predicted loci, of which 83% are assigned a functional annotation. We leverage the genomes and transcriptomes of 38 additional plant species to assign Ceratopteris predicted proteins to 17,489 orthologous families. Phylogenomic analysis of these gene families identifies dynamic gain, loss and amplification of different orthogroups in the last common ancestor (LCA) of the major plant lineages, including in the sister lineages of ferns and seed plants. We then characterize the evolutionary history of the GRAS domain proteins, an important superfamily of regulatory proteins with a complex evolutionary history (Cheng et al. 2019). GRAS proteins were assigned to 15 orthologous gene families with varying taxonomic representation. We further refine these orthogroups into 19 subfamilies, including one (GRAS-I) that is ubiquitous in ferns and absent in most seed plants, including the model plant *Arabidopsis thaliana* (hereafter Arabidopsis). Taken together, the results of our study illustrate the utility of our Ceratopteris transcriptome, which will be a valuable genetic resource to facilitate molecular, evolutionary, and genetic studies in ferns.

## Materials and Methods

### Plant Materials and Growth Conditions

**Fig. 1.**
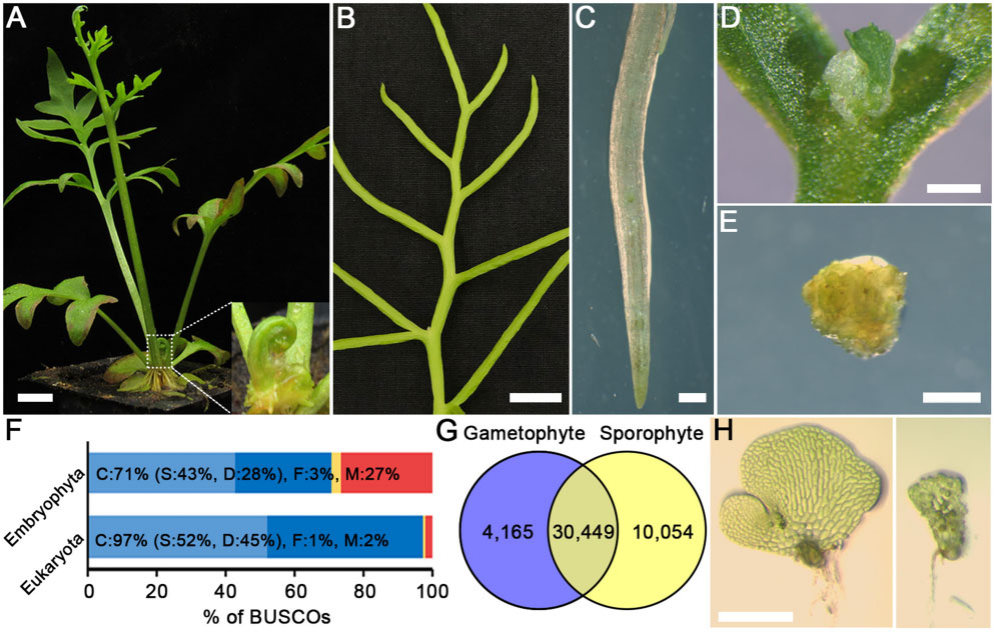
Representative images of *Ceratopteris richardii* sporophyte tissues used for the RNA sequencing and summary of transcriptome annotations. (*A*–*E*) Shoot apex (*A*, inset); fertile frond (*B*); root (*C*); bulbil (*D*); and callus (*E*) generated from sporophyte tissues. Scale bars = 1 cm (*A* and *B*), 1 mm (*C*–*E*). (*F*) BUSCO completeness assessments of the *Ceratopteris* transcriptome with the Embryophyta dataset_odb9, *n* = 1,440 and Eukaryota dataset_odb9, *n* = 303. Blue, yellow and red bars, respectively represent the proportion of complete (C), fragmented (F), and missing (M) BUSCOs. Light blue bars represent complete and single-copy BUSCOs (S). Dark blue bars represent complete and duplicated BUSCOs (D). (*G*) Venn diagram showing the number of genes commonly and uniquely detected between the gametophyte and sporophyte tissues. (*H*) Hermaphroditic (left) and male (right) gametophytes at 11 days after inoculation. Scale bar = 0.5 mm.

The Ceratopteris accession Hn-n (Hickok et al. 1987) was used in this study. Sporophytes were generated by self-fertilizing wild-type hermaphroditic gametophytes grown on FM plates containing 0.7% (w/v) agar (Sigma–Aldrich). Once sporophytes developed roots, they were transferred to soil. Young sporophytes were grown at 28 °C, under continuous light. Adult sporophytes were grown at 28 °C in greenhouse at Purdue. Five sporophyte tissues (fig. 1*A*–*E*) were harvested for RNA isolation. Shoot apices (fig. 1*A*) and roots (fig. 1*C*) were harvested from young sporophytes. Fertile fronds (fig. 1*B*) and bulbils (fig. 1*D*) were harvested from adult sporophytes. Calli were induced from shoot tips or fronds of young Ceratopteris sporophytes (∼3 weeks old) on the callus induction plates (pH 5.8) containing 1× MS salts (PhytoTechnology Laboratories), 2% (w/v) sucrose and 1 mg l^−1^ benzylaminopurine (BAP), and 0.7% agar (Sigma–Aldrich). Fully induced calli (fig. 1*E*) were collected for RNA isolation. Gametophyte sample preparation and RNA-seq are described previously (Atallah et al. 2018).

### RNA Isolation, Library Construction and Sequencing

Total RNAs were isolated using the RNeasy Mini Kit (Qiagen). TruSeq DNA PCR-Free Low Throughput Library Prep Kit (Illumina) was used to construct cDNA libraries from each sample except for bulbil and root samples. Due to relatively low amount of RNA in the bulbil and root samples, the Universal Plus mRNA Kit (NuGen) was used to construct cDNA libraries for these tissues. All libraries were sequenced on the NovaSeq 6000 Sequencing System (Illumina). Library construction and sequencing were performed at the Purdue Genomics Core Facility. The gametophyte RNA-seq data were acquired previously (Atallah et al. 2018).

### De Novo Transcriptome Assembly and Annotation

Illumina reads containing erroneous k-mers were detected by rCorrector (Song and Florea 2015) and removed. Adapters and low-quality bases were trimmed using Trim Galore! (v0.4.4) and cutadapt (v1.13) (Martin 2011) with flags “--paired --phred33 --length 36 -q 5 --stringency 1 -e 0.1.” Quality trimmed reads were mapped using bowtie2 (v2.3.3.1) to the SILVA database (SILVA_132_NR99) (Quast et al. 2013), the Ceratopteris chloroplast genome (KM052729.1), and the Arabidopsis mitochondrion genome (NC_001284.2) to remove rRNA, chloroplast and mitochondria DNA contamination, respectively. A de novo transcriptome was assembled from the cleaned reads (supplementary table S1, Supplementary Material online) using Trinity (v2.6.6) (Grabherr et al. 2011) using default settings with the strand-specific method enabled. Each contig is defined as an isoform; a Trinity gene is defined as a cluster of similar contigs predicted to arise from the same locus in the genome.

Predicted coding regions were identified using TransDecoder (v3.0.1) (Haas et al. 2013), and contigs that lacked any predicted peptide were removed from the final transcriptome. To reduce the number of spurious coding regions, the transcriptome was run through the Trinotate pipeline (v3.1.1) (Bryant et al. 2017). Briefly, potential homologs of predicted Ceratopteris peptide sequences were identified using BLASTx and BLASTp (DIAMOND, v0.8.36) (Buchfink et al. 2015) to query the NCBI refseq database (retrieved June 2018) and the Araport11 database, respectively. Coding regions without a significant hit to either database (*E*-value ≤ 1 × 10^−8^) were excluded from the final transcriptome. Functional annotations were assigned via InterProScan (v5.29-68.0) (Jones et al. 2014).

We checked the transcriptome for possible contamination using the Alien Index (AI) pipeline (https://github.rcac.purdue.edu/jwisecav/phylo-pipe; last updated August 26, 2019) as previously described (Wisecaver et al. 2016; Gonçalves et al. 2018). Briefly, each predicted protein sequence was queried against the NCBI RefSeq database (retrieved June 2018) using DIAMOND (v0.8.36) (Buchfink et al. 2015), and the AI score was calculated based on the output. The AI score is given by the formula: AI=*nbsO*-*nbsV*, where *nbsO* is the normalized bit score of the best hit to a species outside of the eudicot lineage, *nbsV* is the normalized bit score of the best hit to a species within the Viridiplantae lineage (i.e., green plants and algae). AI scores range from –1 to 1, being greater than zero if the predicted protein sequence had a better hit to a nonviridiplantae species, suggestive of either horizontal gene transfer (HGT) or contamination (Wisecaver et al. 2016; Gonçalves et al. 2018). Although HGT is a potential source of alien genes and has been documented in ferns (Li et al. 2014), it is difficult to differentiate between true HGT and contamination in a de novo transcriptome due to the inability to confirm true integration of a foreign gene into the host nuclear genome. Therefore, proteins with an AI score ≥ 0.05 were considered possible contaminants from other species and excluded from downstream analyses. In addition, contigs matching sequences from the Ceratopteris chloroplast genome, Arabidopsis mitochondrial genome, and SILVA database (for rRNA) detected using BLASTn (BLAST+, v2.7.1; *E*-value ≤ 10) were also excluded. Lastly, a BUSCO (v2.0) analysis was performed to assess the completeness of the final transcriptome using the eukaryote_odb9 (303 conserved genes) and embryophyta_odb9 (1,440 conserved genes) data sets (Simão et al. 2015).

### Identification and Analysis of Homologous Gene Families

Homology between the predicted proteomes of Ceratopteris and 38 other plant species (supplementary table S2, Supplementary Material online) was determined with OrthoFinder (v2.1.2) (Emms and Kelly 2015) with sequence similarity searches performed by DIAMOND (Buchfink et al. 2015), alignments using MAFFT v7.407 (Katoh and Standley 2013), and tree building with FastTree v2.1.7 (Price et al. 2010).

Orthogroup evolutionary changes including gains, losses, expansions and contractions were inferred with the program Count (Csűös 2010) using Dollo parsimony and unweighted Wagner parsimony. In both types of parsimony analyses, an orthogroup gain is defined as a shift from orthogroup absence at the preceding node to presence at the node of interest; whereas, orthogroup loss is the opposite transition. Under Dollo parsimony an orthogroup may be lost multiple times but gained only once. This approach effectively pushes any gain back in time to the LCA of any species present in that orthogroup. Therefore, Dollo parsimony is the more conservative estimator of orthogroup gain at leaf nodes as well as interior nodes that are closer to the leaves. Conversely, a Wagner parsimony approach, in which gene gain and gene loss are weighted the same, is a more conservative method for estimating gene gain for nodes closer to the root of the species tree. For example, when dealing with a hypothetical orthogroup that is present in Ceratopteris and the chlorophyte *Chlamydomonas reinhardtii* yet absent from all other species, Dollo parsimony would predict that the orthogroup was gained in the LCA of these two species (corresponding to the root of the species tree) and subsequently lost multiple times. In contrast, Wagner parsimony would predict that the orthogroup was gained independently in the two species, requiring no subsequent losses. One biological explanation for an orthogroup being acquired independently in different species is HGT; however, HGT appears to be relatively rare in land plants. Methodological artifacts in the OrthoFinder prediction (which would cause genes to be assigned to orthogroups incorrectly) could also result in an orthogroup being gained multiple times in our analysis under Wagner parsimony. For these reasons, the two parsimony methods represent realistic bounds for estimating the number of gene changes at a node.

In addition to comparing orthogroup gain and loss, we also investigated how orthogroup copy number evolved across the species tree. Using Wagner parsimony, orthogroup expansion is defined as a shift from a single-copy state at the preceding node to a multi-copy state at the node of interest; whereas, an orthogroup contraction is the shift from multi-copy to single-copy. Changes in copy number were also inferred from the OrthoFinder analysis directly; duplications with clade support ≥ 0.50 were parsed from the OrthoFinder duplications.csv output.

GO annotations for Arabidopsis and Ceratopteris were assigned via InterProScan (v5.29-68.0) (Jones et al. 2014). Tests for functional enrichment were performed in R Bioconductor v3.11 by first creating custom orgDBs using AnnotationForge v1.30.1. GO enrichment tests were then performed by clusterProfiler v3.16.1 using the function enrichGO. *P*-values were adjusted for multiple comparisons using the Benjamini & Hochberg (BH) method. Network maps of enriched terms were also created by clusterProfiler using the function emapplot.

### Phylogenetic Analysis of GRAS Family Proteins

We performed separate phylogenetic analyses for all the orthogroups containing sequences that were annotated by InterProScan with the GRAS domain (PF03514). Sequences in each orthogroup were aligned with MAFFT (v7.407) using the E-INS-I strategy and following parameters: --maxiterate 1000 --bl 30 --op 1.0 --retree 1 (Katoh and Standley 2013). Maximum likelihood trees were constructed using IQ-TREE (v1.6.10) (Nguyen et al. 2015) using the built in ModelFinder to determine the best-fit substitution model (Kalyaanamoorthy et al. 2017) and performing SH-aLRT and ultrafast bootstrapping analyses with 1,000 replicates each. Following initial tree building, several sequences were excluded due to their long branch lengths, including Ginko_biloba_GBI00007557 and Ginko_biloba_GBI00011220 in OG0001609, Amborella_trichopoda_ATR0582G205 and Populus_trichocarpa_Potri.004G208700 in OG0003235, Polytrichum_commune_1kpSZYG2036234 in OG0005632, Picea_abies_PAB00030745 in OG0007617, Sceptridium_ dissectum_1kpEE in OG0007977, and Amborella_trichopoda_ATR0174G041 in OG0010265. Alignments and phylogenetic trees were reconstructed following sequence curation. Trees were visualized and annotated using iTOL (v4) (https://itol.embl.de/itol.cgi, last accessed March 9, 2021) (Letunic and Bork 2019). The distribution of GRAS proteins was displayed using TBtools (Chen et al. 2020).

## Results

### The *Ceratopteris richardii* Transcriptome

To generate a comprehensive transcriptome of Ceratopteris, independent RNA libraries from two gametophyte tissues (males and hermaphrodites) (Atallah et al. 2018) and five sporophyte tissues (shoot apices, fertile fronds, roots, bulbils, and calli) (fig. 1*A*–*E*) were generated and sequenced resulting in ∼2.6 billion cleaned paired-end reads (supplementary table S1, Supplementary Material online). The de novo assembly consisted of 64,974 contigs with an average GC content of 43.04% and an N50 of 2,704 bp. Assembly contigs were collapsed into 44,668 Trinity genes, which are defined by the Trinity assembler as clusters of similar contigs predicted to arise from the same locus in the genome (Grabherr et al. 2011) and are hereafter referred to simply as genes. A BUSCO analysis (Simão et al. 2015) was performed to evaluate the completeness of the Ceratopteris transcriptome, recovering 274 of the 303 conserved eukaryotic genes (97%) and 1,023 of 1,440 conserved embryophyta genes (71%) (fig. 1*F*). This result is comparable to the similar BUSCO analysis that was conducted using the transcriptome of another homosporous fern, *Polypodium amorphum*, which recovered 1,028 of 1,440 conserved embryophyta genes (71%) (Sigel et al. 2018).

Functional annotations were assigned to the majority (83%) of predicted proteins. In total, 27,029 (60.5%) of genes were assigned at least one Pfam domain (El-Gebali et al. 2019). Gene Ontology (GO) (Harris et al. 2004) associations were also common, with 21,481 (48.1%) of genes assigned to at least one GO category. Additional functional annotations were assigned to 67.5%, 51.4%, and 6.1% of genes using the PANTHER (Mi et al. 2013), InterProScan (Jones et al. 2014), and KEGG (Kanehisa et al. 2008) databases, respectively.

Within the Ceratopteris transcriptome, 68.2% (30,449) of the total genes are expressed within both gametophyte and sporophyte tissues (fig. 1*G* and *H*). In contrast, in the moss *Physcomitrella patens* (a species with a gametophyte-dominate life cycle), 85.5% of genes are detected in both gametophyte and sporophyte phases (Ortiz-Ramirez et al. 2016). In the angiosperm Arabidopsis, only 30.6% of genes are shared in both sperm cells and seedlings (Borges et al. 2008). These data demonstrate a trend of decreased number of genes commonly expressed in both gametophyte and sporophyte stages and is consistent with the highly reduced gametophytes in seed plants and the transition from gametophyte-dominant to sporophyte-dominant life cycles.

**Fig. 2.**
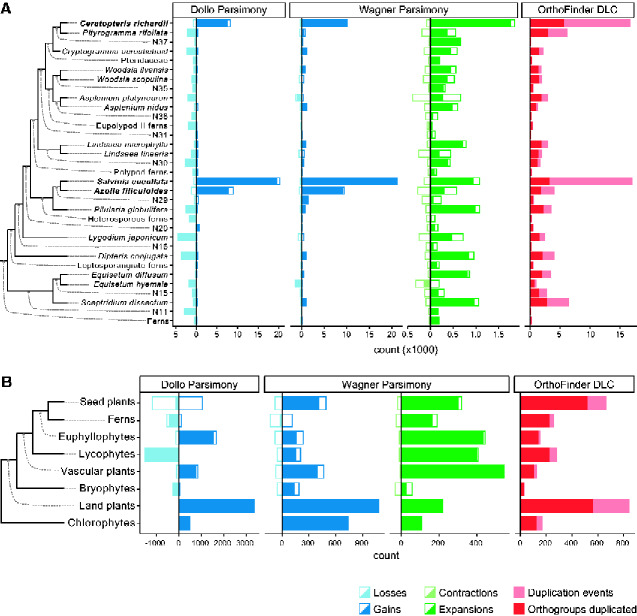
Gene family evolutionary dynamics across plants. Ancestral gene content reconstruction of OrthoFinder orthogroups (*A*) within ferns and (*B*) at each of the major interior nodes of the green plant species tree. For both Dollo and Wager parsimony analyses, unfilled bars and numbers to the right of the vertical axes represent the number of gene families gained/expanded at a node relative to its parent node. Bars and numbers to the left of the vertical axes represent the number of gene families lost/contracted. The filled bars represent the net effect, either gain or loss as well as either expansion or contraction of gene families, at each node. For the OrthoFinder DLC analysis, the pink bars represent the total number of duplication events predicted to have occurred at each node, and the red bars represent the number of orthogroups that duplicated (one or more times). Results for all species and interior nodes are available in supplementary table S3, Supplementary Material online.

To investigate the evolutionary dynamics of plant gene families, we performed an OrthoFinder analysis (Emms and Kelly 2015) using the predicted proteome of Ceratopteris and 38 additional genomes and transcriptomes from across the Viridiplantae (fig. 2, supplementary table S2, Supplementary Material online). Included in our analysis were genomes from the two heterosporous ferns *S. cucullata* and *A. filiculoides* as well transcriptome sequences from 14 additional ferns produced by the oneKP (Leebens-Mack et al. 2019). The OrthoFinder analysis identified 172,891 orthogroups (predicted gene families), of which 22,919 were present in two or more species in the analysis (supplementary note S1, Supplementary Material online). The average number of orthogroups assigned to species with genome-level data (i.e., excluding the oneKP and Ceratopteris transcriptomes) was 15,496 and ranged from 8,066 in the chlorophyte *Micromonas commode* to 29,456 in the fern *A. filiculoides* (supplementary table S2, Supplementary Material online); Ceratopteris proteins, despite being predicted from a de novo transcriptome, were assigned to a comparable number of orthogroups (*n* = 17,489). The number of orthogroups that were gained in (i.e., unique to) Ceratopteris was large (Dollo *n* = 8,320, Wagner *n* = 10,140, fig. 2*A*, supplementary table S3, Supplementary Material online) as was the number of species-specific amplifications (expansions *n* = 1,848, duplications *n* = 16,635, fig. 2*A*, supplementary table S3, Supplementary Material online). These numbers are comparable to those seen in the two ferns with sequenced genomes (fig. 2*A*, supplementary table S3, Supplementary Material online).

### Gene Family Evolution in the Euphyllophyte LCA

The OrthoFinder gene families were further analyzed to identify genes that were gained, amplified or lost in the LCA of the major plant lineages (fig. 2*B*, supplementary table S3, Supplementary Material online), focusing particularly on the euphyllophyte sister lineages of ferns and seed plants. The LCA of euphyllophytes showed a net trend of more orthogroups gained than lost (Dollo *n* = 1,527; Wagner *n* = 159; fig. 2*B*, supplementary table S3, Supplementary Material online). Arabidopsis genes that were gained at this internode were enriched in 21 and 48 GO terms under Dollo and Wagner parsimony, respectively (Benjamini–Hochberg adjusted *P-*value < 0.05). GO terms recovered under either method included those involved in synthesis of cell walls, gene regulation, as well as lipid transport and localization (supplementary table S4, Supplementary Material online).

**Fig. 3.**
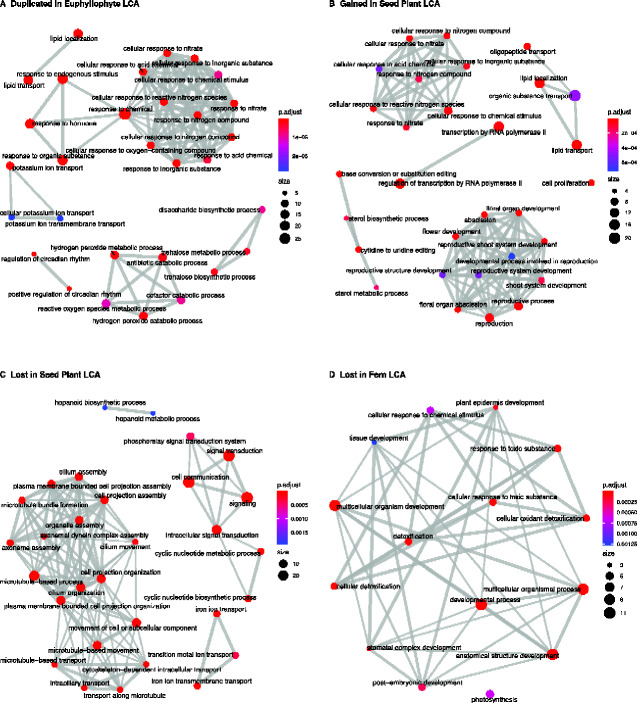
GO enrichment network graphs for exemplar gene sets. Enrichment maps for genes (*A*) duplicated in the euphyllophyte LCA, (*B*) gained or (*C*) lost in the seed plant LCA, and (*D*) lost in the fern LCA. Enriched terms are organized into a network with edges connecting overlapping gene sets. Mutually overlapping gene sets are clustered together, making it easier to identify functional modules.

A moderate number of duplication events were also predicted at the euphyllophyte LCA (*n* = 136; fig. 2*B*, supplementary table S3, Supplementary Material online). Arabidopsis genes that duplicated at this internode were enriched in 59 GO categories (supplementary table S4, Supplementary Material online), which clustered in several functional groups. The largest cluster includes GO terms that appeared to be involved in response to nitrogen (fig. 3*A*); however, closer inspection of the genes in this set suggests they encode membrane transporters and membrane channels more generally, including cyclic GMP activated Ca2+ channels as well as porins and anion transporters (supplementary table S4, Supplementary Material online). Genes that participate in metabolic or cellular processes, including peroxidases, trehalose-phosphatase/synthases, and Sec23/Sec24 protein transport family proteins form a second cluster of enriched GO terms (fig. 3*A*, supplementary table S4, Supplementary Material online). Other clusters of GO terms are associated with developmental processes including regulators of circadian rhythm and responses to various stimuli (fig. 3*A*). All genes represented by the circadian rhythm cluster are *EARLY FLOWERING 4* (*ELF4*) or *ELF4*-like genes (supplementary table S4, Supplementary Material online). The cluster of GO terms involved in response to stimuli are defined by genes involved in auxin responses and include *NON-PHOTOTROPHIC HYPOCOTYL* and *AUXIN RESPONSE FACTORS* (*ARF*)*—MONOPTEROS* (*ARF5*) and *ETTIN* (*ARF3*) (supplementary table S4, Supplementary Material online).

### Gene Family Evolution in the Seed Plant LCA

The results for the seed plant LCA were mixed, with Dollo parsimony showing a net loss of orthogroups (*n* = 128) and Wagner parsimony indicating a net gain (*n* = 411) (fig. 2*B*). Here we focus on the GO terms enriched in the Dollo gene sets; the results for Wagner are available in supplementary table S4, Supplementary Material online. Arabidopsis genes that were gained at this internode were enriched in 44 GO terms (supplementary table S4, Supplementary Material online), which clustered into several functional categories including reproduction, response to nitrogen, lipid and sterol biosynthesis, and transcription regulation (fig. 3*B*). Manual inspection of the genes in this GO category suggests that these patterns are driven by a handful of gene families including *INFLORESCENCE DEFICIENT IN ABSCISSION* (*IDA*; involved in reproduction), *GIRDIN* (involved in response to nitrogen), *PSK* (cell proliferation), *SQUALENE MONOOXYGENASE* (sterol biosynthesis), and *bHLH* DNA-binding genes (transcription regulation) (supplementary table S4, Supplementary Material online). An additional set of GO terms involved in cell fate and signaling were significantly enriched; however, the two gene set was not large enough to be displayed in figure 3*B*. This cell fate and signaling gene set was defined by the *CLAVATA3/ESR-RELATED 25* (*CLE25*) gene family (supplementary table S4, Supplementary Material online). The acquisition of the *CLE25* genes in seed plants is well documented and indicates an evolutionary step in the regulation of transpiration (Takahashi et al. 2018). In Arabidopsis, the CLE25 peptide regulates several processes, including the regulation of phloem initiation (Ren et al. 2019) as well as inducing stomata closure through modulation of abscisic acid accumulation (Takahashi et al. 2018).

In addition to gene gain, a large number of lineage-specific gene amplifications occurred in the LCA of seed plants (expansions *n* = 320, duplications *n* = 665; fig. 2*B*; supplementary table S3, Supplementary Material online). Arabidopsis genes that duplicated at this internode were enriched in 111 GO terms (Supplementary table S4, Supplementary Material online). As to be expected, MADS box transcription factors that regulate floral organ identity in flowering plants were recovered in this gene set (supplementary table S4, Supplementary Material online). Other enriched GO terms associated with development are defined by genes involved in apical-basal patterning of the embryo (GNOM), photomorphogenesis (phytochrome and NPH3), and epigenetic regulation of gene expression (SU(VAR)3-9 homolog proteins) (supplementary table S4, Supplementary Material online). Arabidopsis genes that expanded from single to multi-copy in the seed plant LCA (as predicted by Wagner parsimony; see Materials and Methods) were enriched in 90 GO terms, the majority of which involve metabolic and cell biology processes (supplementary table S4, Supplementary Material online). Cell wall and pectin biogenesis and modifying genes form one large cluster of enriched GO terms and include COBRA, pectin lyases, glucuronoxylan methyltransferases and xyloglucan: xyloglucosyl transferases (supplementary table S4, Supplementary Material online). In agreement with our analysis, the radiation of the COBRA gene family, which is necessary for oriented cell expansion in Arabidopsis, has previously been noted (Sorek et al. 2016). All enriched GO terms related to amino acid/anion transport are attributed to the GLUTAMINE DUMPER gene family (supplementary table S4, Supplementary Material online). Lastly, the enrichment GO terms involved in nitrate response was driven by the HT1 protein kinase, which regulates stomata opening in response to red-light and CO_2_ (Matrosova et al. 2015).

To evaluate the types of gene functional categories that were lost in the seed plant LCA (and therefore absent in Arabidopsis), we performed GO term enrichment analyses on the Ceratopteris genes that were present in these orthogroups, identifying 42 and 37 enriched GO terms in the Dollo and Wagner analyses, respectively (supplementary table S4, Supplementary Material online). Here again, we focus on the Dollo gene set, in which the largest cluster of enriched GO terms were involved in development of flagellated cells (fig. 3*C*, supplementary table S4, Supplementary Material online). The loss of gene families required for the flagella in the seed plant LCA is consistent with the absence of flagella in the sperm cells of angiosperms and most gymnosperms (Rudall and Bateman 2007). Also enriched in this gene set were GO terms involved in hopanoid triterpenoid biosynthesis (fig. 3*C*, supplementary table S4, Supplementary Material online). This pattern was driven by one gene family (OG0007870), which was present in two bryophytes and 10 ferns including Ceratopteris (supplementary fig. S1, Supplementary Material online). The sparse distribution of this gene family in land plants is consistent with the previous report (Li et al. 2018), and these findings could be suggestive of HGT, a process that has already been speculated to play a role in the evolution of triterpenoid synthases in ferns (Frickey and Kannenberg 2009; Li et al. 2018).

### Gene Family Evolution in the Fern LCA

The fern LCA showed a net trend of more orthogroups lost than gained (Dollo *n* = 429; Wagner *n* = 18; fig. 2*B*, supplementary table S3, Supplementary Material online). However, we are cautious not to overinterpret a trend of gene loss at this internode as all but two species of ferns are represented by transcriptome data only. Genes may be absent from a de novo transcriptome if they show little to no expression in the sampled tissues and will therefore appear lost in our analysis. Moreover, because of the evolutionary distance between Ceratopteris and model seed plants, assigning GO terms based on homology is also less accurate.

We did not identify any enriched GO terms in the Ceratopteris gene sets that were gained at this internode under either Dollo or Wagner parsimony. Although the number of gene families gained in the fern LCA was low, there was a moderate number of lineage-specific gene amplifications at this internode (expansions *n* = 190, duplications *n* = 255; fig. 2*B*, supplementary table S3, Supplementary Material online). Ceratopteris genes that duplicated in the fern LCA were enriched in 76 GO terms, including those involved in response to stimuli, fatty acid biosynthesis, and cell wall organization (supplementary table S4, Supplementary Material online). Ceratopteris genes that expanded from single to multi-copy in the fern LCA were enriched in 85 GO terms, the majority of which involve jasmonic acid metabolism, regulation of cytokinesis, and photoperiodism (supplementary table S4, Supplementary Material online).

To evaluate the types of gene functional categories that may have been lost in the fern LCA (and therefore absent in Ceratopteris), we performed GO term enrichment analyses on the Arabidopsis genes that were present in these orthogroups, identifying 15 and 52 enriched GO terms in the Dollo and Wagner analyses, respectively (supplementary table S4, Supplementary Material online). Focusing on the Dollo results, the enriched GO terms in this gene set include photosynthesis and a variety of related categories broadly involved in tissue and organ development (fig. 3*D*). Enrichment of the photosynthesis GO terms was driven by five genes that encode proteins that are components of photosystem I and II (supplementary table S4, Supplementary Material online). The rest of the enriched GO terms were made up of *YABBY* transcription factors, *EPIDERMAL PATTERNING FACTOR* genes, and *RESPONSE TO LOW SULFUR* genes (supplementary table S4, Supplementary Material online).

### Phylogenetic Analysis of GRAS Domain Proteins

**Fig. 4.**
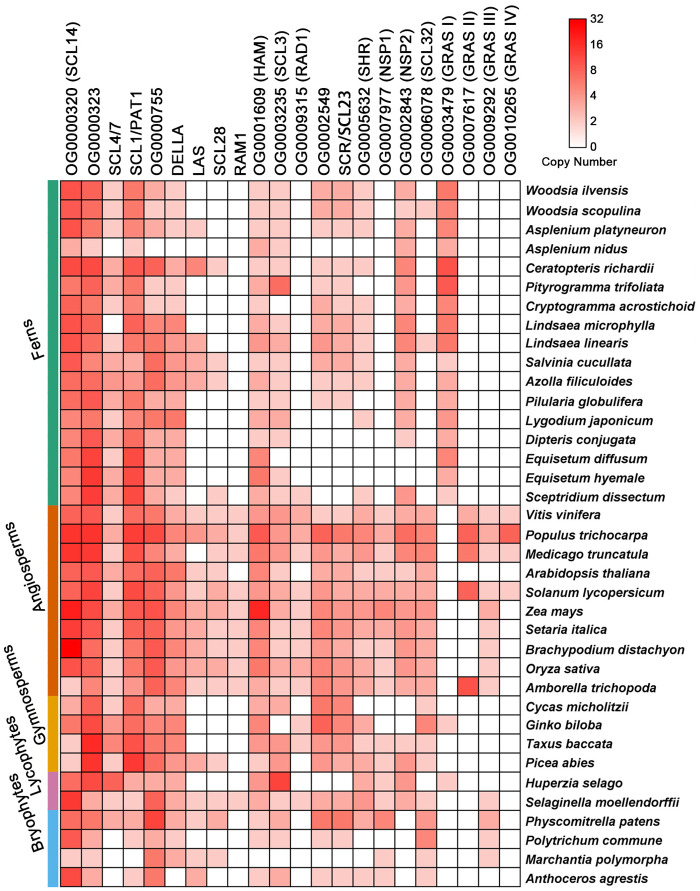
A heatmap showing the distribution of GRAS proteins in 37 land plant species. The copy number of GRAS proteins in each species was determined by phylogenetic analyses (supplementary figs. S3–S17, Supplementary Material online). The left color-coded bars indicate the taxonomic classification of each plant species. Green: ferns; orange: angiosperms; yellow: gymnosperms; purple: lycophytes; and blue: bryophytes. GRAS members were clustered into 15 orthogroups including at least 19 distinct subfamilies. Subfamilies were named after their founding member identified in Arabidopsis or Medicago, with the exception of four uncharacterized subfamilies that were named with Roman numerals. The SCL4/7 and SCL1/PAT1 subfamilies belong to OG0000323; the DELLA, LAS, SCL28, and RAM1 subfamilies belong to OG0000755; and the SCR/SCL23 subfamily belongs to OG0002549. The copy number was quantitatively indicated by color with the scale from white (0) to red (32).

To further illustrate the utility of our gene family analysis, we selected the GRAS domain proteins to investigate in more detail. This superfamily of proteins is thought to have evolved as crucial regulators in control of various plant growth and developmental processes, including shoot and root development, stem cell homeostasis, light and hormone signaling, responses to biotic and abiotic stresses, and symbiosis with microorganisms (Hirsch and Oldroyd 2009; Bolle 2016). GRAS proteins clustered into 15 orthogroups that were variably present in the different plant lineages (fig. 4). To better understand the evolutionary history of GRAS proteins, maximum-likelihood phylogenetic trees were built for each orthogroup (supplementary figs. S2–S16 and supplementary notes S2–S16, Supplementary Material online). OG0000320 was one of two fully conserved GRAS-containing orthogroups present in all species in the analysis (fig. 4). This orthogroup contained the functionally characterized Arabidopsis GRAS homolog AtSCL14 (AT1G07530) (supplementary fig. S2, Supplementary Material online), which is likely involved in the detoxification of xenobiotics (Fode et al. 2008). The second orthogroup containing all species in the analysis was OG0000323, which consisted of two subfamilies, with one subfamily containing the Arabidopsis GRAS homologs AtSCL4 (AT5G66770) and AtSCL7 (AT3G50650) and the second subfamily containing the Arabidopsis GRAS homologs AtSCL1 (AT1G21450) and AtPAT1 (AT5G48150) (supplementary fig. S3, Supplementary Material online).

Three orthogroups (OG0000755, OG0001609, and OG0003235) were present in all lineages and the majority of species in the analysis (fig. 4). OG0000755, a large orthogroup with 171 sequences, was subdivided into four major subfamilies (fig. 4, supplementary fig. S4, Supplementary Material online). OG0000755 was the only GRAS orthogroup that contained a significant number of multi-domain proteins; 34 proteins in this orthogroup were annotated with the DELLA Pfam domain (PF12041) in addition to GRAS (PF03514) (supplementary fig. S4, Supplementary Material online). The majority of DELLA-containing proteins in this orthogroup were from angiosperms, including the five Arabidopsis DELLA proteins AtRGL1 (AT1G66350), AtRGL2 (AT3G03450), AtRGL3 (AT5G17490), GAI (AT1G14920), and RGA (AT2G01570). In Arabidopsis DELLA proteins function as crucial repressors in the gibberellin signaling pathway (Vera-Sirera et al. 2016). DELLA domains were also identified in two Ceratopteris sequences (fig. 4, supplementary fig. S4, Supplementary Material online), suggesting ferns and seed plants share conserved components to transduce the gibberellin signal. A second subfamily within the larger OG0000755 orthogroup contained three sequences with shared conserved roles in control of axillary bud initiation and lateral shoot development (Schumacher et al. 1999; Greb et al. 2003; Li et al. 2003): LATERAL SUPPRESSOR (LAS) in Arabidopsis (AT1G55580) (Greb et al. 2003); MONOCULM 1 (MOC1) in *Oryza sativa* (Os06g40780) (Li et al. 2003); and Lateral suppressor (LS) in *Solanum lycopersicum* (Solyc07g066250.1) (Schumacher et al. 1999) (fig. 4, supplementary fig. S4, Supplementary Material online). A third subfamily in OG0000755 is present in at least one species in each of the five plant lineages in our analysis and is fully present in all angiosperms, including Arabidopsis AtSCL28 (AT1G63100). Lastly, OG0000755 contained one more characterized GRAS protein from *Medicago truncatula* (hereafter Medicago) (RAM1), which participates in the symbiosis with arbuscular mycorrhizal fungi (Gobbato et al. 2012). RAM1 homologs are present in all angiosperm genomes with the notable exception of Arabidopsis (fig. 4, supplementary fig. S4, Supplementary Material online). Outside of the angiosperms, the RAM1 subfamily appears to group closely with one additional sequence from the lycophyte *Selaginella moellendorffi* and appears to be absent in the bryophytes, ferns, and gymnosperms in this analysis (fig. 4, supplementary fig. S4, Supplementary Material online).

OG0001609 consisted of the HAIRY MERISTEM (HAM) family, including the Arabidopsis HAM homologs—AtHAM1 (AT2G45160), AtHAM2 (AT3G60630), AtHAM3 (AT4G00150), and AtHAM4 (AT4G36710) (supplementary fig. S5, Supplementary Material online). HAM proteins control the determinacy and proliferation of shoot apical meristems and the de novo formation of axillary meristems in petunia and Arabidopsis (Stuurman et al. 2002; Schulze et al. 2010; Engstrom et al. 2011; David-Schwartz et al. 2013; Fan et al. 2015; Zhou et al. 2015; Hendelman et al. 2016; Zhou et al. 2018; Han, Liu, et al. 2020; Han, Geng, et al. 2020). Members of the HAM subfamily are present in almost all the species in the analysis, with the exception of the liverwort *Marchantia polymorpha* (fig. 4). Although taxon sampling is different, the topology of the HAM phylogenetic tree in this study is generally in agreement with the result we showed previously (Geng et al. 2021). Lastly, OG0003235 contained the Arabidopsis GRAS homolog AtSCL3 (AT1G50420) (supplementary fig. S6, Supplementary Material online), which antagonizes the DELLA proteins and regulates the gibberellin signaling pathway in Arabidopsis (Zhang et al. 2011).

Six orthogroups (OG0009315, OG0002549, OG0005632, OG0007977, OG0002843, and OG0006078) were well represented in angiosperms but variably present in the other four lineages (fig. 4). OG0009315 contained the Medicago protein RAD1, which, similar to RAM1 in OG0000755, is involved in the symbiosis with arbuscular mycorrhizal fungi (Rey et al. 2017). Also like the RAM1 subfamily, RAD1 is conspicuously absent in Arabidopsis, despite being found all other angiosperms in the analysis (fig. 4, supplementary fig. S7, Supplementary Material online). OG0002549 (supplementary fig. S8, Supplementary Material online) and OG0005632 (supplementary fig. S9, Supplementary Material online) together contain three functionally characterized proteins in Arabidopsis: SCR (AT3G54220), SCL23 (AT5G41920), and SHR (AT4G37650), which collectively determine both the root and shoot radial patterning (Cui et al. 2007; Long et al. 2015; Yoon et al. 2016). Both SCR and SCL23 belong to OG0002549 and they are derived from a duplication in the LCA of seed plants (supplementary fig. S8, Supplementary Material online). OG0007977 and OG0002843 contain NSP1 (supplementary fig. S10, Supplementary Material online) and NSP2 (supplementary fig. S11, Supplementary Material online) proteins, respectively. NSP1 and NSP2 were first characterized in Medicago (Kaló et al. 2005; Smit et al. 2005) where they are involved in nodulation and strigolactone biosynthesis (Kaló et al. 2005; Smit et al. 2005; Liu et al. 2011). These two orthogroups are present in the majority of angiosperms and lycophytes, but display divergent distributions in ferns and bryophytes (fig. 4). Members of OG0007977 are absent in all ferns in our analysis but present in several bryophytes. In contrast, members of OG0002843 are largely retained in ferns but are absent in all the species of bryophytes. Lastly, OG0006078 contains the Arabidopsis GRAS homolog AtSCL32 (AT3G49950) and members from this orthogroup are widely present in examined species with the exception of ferns (fig. 4, supplementary fig. S12, Supplementary Material online).

The four remaining orthogroups (OG0003479, OG0007617, OG0009292, and OG0010265) are uncharacterized and absent from the majority of species in our analysis, including Arabidopsis (fig. 4, supplementary figs. S13–S16, Supplementary Material online). OG0003479 was the most intriguing as this orthogroup as it was present in all 17 ferns and completely absent in angiosperms (fig. 4, supplementary fig. S13, Supplementary Material online).

## Discussion

### A Ceratopteris Transcriptome Database Provides a Platform for Functional Genomic Studies in Ferns

To explore the protein coding gene space and to generate a comprehensive catalog of transcripts in Ceratopteris, 17 independent sequencing libraries from gametophyte and sporophyte tissues were sequenced at great depth (∼2.6 billion cleaned paired-end reads) in total (supplementary table S1, Supplementary Material online) prior to de novo assembly. The BUSCO analysis showed that this transcriptome covers 97% eukaryotic genes and 71% of conserved embryophyta genes. We also performed OrthoFinder analysis (Emms and Kelly 2015) using this Ceratopteris transcriptome plus 38 additional genomes and transcriptomes from species across the Viridiplantae. 17,489 orthogroups were assigned to Ceratopteris, which is comparable to the number of orthogroups identified from the published genomes of two aquatic ferns, *S. cucullata* (*n* = 17,593) and *A. filiculoides* (*n* = 29,456) (supplementary table S2, Supplementary Material online) (Li et al. 2018). Among them, the GRAS domain homologs from the Ceratopteris transcriptome and the two aquatic fern genomes are consistently present or absent in each orthogroup, and they are clustered together with most GRAS homologs identified from transcriptomes of 14 additional ferns. Collectively, our data indicate that the do novo assembled Ceratopteris transcriptome is accurate and likely comprehensive.

Ceratopteris is a member of the Polypodiales, the most species-rich clade in the fern lineage (PPG I 2016), which retains many characteristics representative of ferns (e.g., homospory and extremely large genomes). The LCA between the Polypodiales and *S. cucullata* and *A. filiculoides* (the two ferns with sequenced genomes) existed over 200 Ma (Kumar et al. 2017), which has allowed for a significant number of accumulated differences between Ceratopteris and our current fern reference genomes (Sessa et al. 2014). Therefore, the incorporation of Ceratopteris, in addition to *S. cucullata* and *A. filiculoides*, in comparative studies of ferns provides tremendous opportunities identifying genes involved the evolution of different developmental or physiological processes within the ferns, including those associated with adaptations to an aquatic environment. These resources are also an entry point for understanding how heterospory arose from homospory within a lineage, which represents a major shift in reproduction (Sussex 1966), and occurred independently in different lineages (Sussex 1966; Bateman and DiMichele 1994).

The Ceratopteris transcriptome and its comparison to other plant genomic resources allowed us to identify genes that were gained or expanded as well as lost or contracted in the LCA of different plant lineages. This analysis is novel in that it leverages the Ceratopteris transcriptome and other recently acquired genome/transcriptome resources from additional ferns to distinguish euphyllophytes from lycophytes and, within the euphyllophyte lineage, ferns from seed plants. The gene gains and losses in the LCA of these lineages are likely to underly the major shifts in plant morphology and physiology that occurred in each of these ancestors, for example, the loss of flagella and shift to sporophyte-dominant lifecycle in seed plants. Due to the difficulty in differentiating true gene loss from missing data in de novo transcriptome assemblies, more genome assemblies for diverse ferns are needed to provide further insight into the dynamics of gene families in land plants.

### The Classification and Evolution of GRAS Domain Proteins in Land Plants

We also analyzed the evolution of the GRAS gene superfamily, which contains crucial regulators in control of various land plant growth and developmental processes (Hirsch and Oldroyd 2009; Bolle 2016). The phylogenies of GRAS proteins from a number of seed plants (Tian et al. 2004; Lee et al. 2008; Engstrom 2011; Wu et al. 2014; Cenci and Rouard 2017; Cheng et al. 2019) and from a few non seed plants (Engstrom 2011; Wu et al. 2014; Cheng et al. 2019) have been characterized. However, the phylogeny of GRAS proteins from homosporous ferns is lacking. In this study, we performed comprehensive phylogenetic analyses of GRAS proteins from 36 species in all the representative land plant lineages including bryophytes, lycophytes, ferns, gymnosperms, and angiosperms. Ferns serve as a very informative middle point in the evolution of land plants. By including several distantly related heterosporous and homosporous ferns in the study, we classified the GRAS domain proteins into 15 orthogroups including at least 19 distinct subfamilies (fig. 4).

Among them, 11 subfamilies (SCL14, SCL4/7, SCL1/PAT1, SCL28, LAS, DELLA, HAM, SCR, SCL3, SHR, and SCL32) contain GRAS homologs from all the representative land plant lineages (fig. 4). These subfamilies include many crucial components in plant growth and development (Hirsch and Oldroyd 2009; Bolle 2016), suggesting that ferns and seed plants may share and exploit similar components to control their body formation. In addition, the sex of the Ceratopteris gametophytes is determined by the antheridiogen (Scott and Hickok 1987; Banks et al. 1993; Banks 1994, 1999), which is one type of gibberellins (Yamane 1998). Since DELLA proteins play important and conserved roles in repressing the gibberellin signaling (Vera-Sirera et al. 2016), the identification of DELLA domain GRAS proteins in Ceratopteris will allow us to perform genetic studies and establish the molecular linkage between the sex determination and gibberellin signaling in ferns in the future.

Members of other four subfamilies—RAM1, RAD1, NSP1, and NSP2 contain essential regulators during the establishment of arbuscular mycorrhizal symbiosis in angiosperms (Delaux et al. 2014, 2015). Despite the wide distribution of the NSP2 subfamily, the other three families (RAM1, RAD1, NSP1) are largely missing in both eusporangiate and leptosporangiate ferns (fig. 4), reflecting the potentially reduced arbuscular mycorrhizal dependency in the fern lineage. Additionally, the GRAS-I subfamily is absent in angiosperms but present in ferns. In contrast, the GRAS-II, GRAS-III, and GRAS-IV subfamilies are present in angiosperms but absent in ferns (fig. 4), suggesting the diversification of these four subfamilies between ferns and angiosperms. Future work to identify the lineage-specific roles of these subfamily members will provide important insights into the evolution of the GRAS domain proteins in land plants.

## Supplementary Material

Supplementary data are available at *Genome Biology and Evolution* online.

## Supplementary Material

evab042_Supplementary_Data
