# Supplementary material for: A De Novo Transcriptome Assembly of Ceratopteris richardii Provides Insights into the Evolutionary Dynamics of Complex Gene Families in Land Plants
Source: Genome Biol Evol. 2021 Mar 3;13(3):evab042. doi: 10.1093/gbe/evab042 (PMC7975763; doi:10.1093/gbe/evab042)
Supplement: evab042_Supplementary_Data [file evab042_supplementary_data.zip › Supplementary information.pdf]

Supplementary information
Including Fig S1-S16, Table S1-S4, and Note S1-S16.

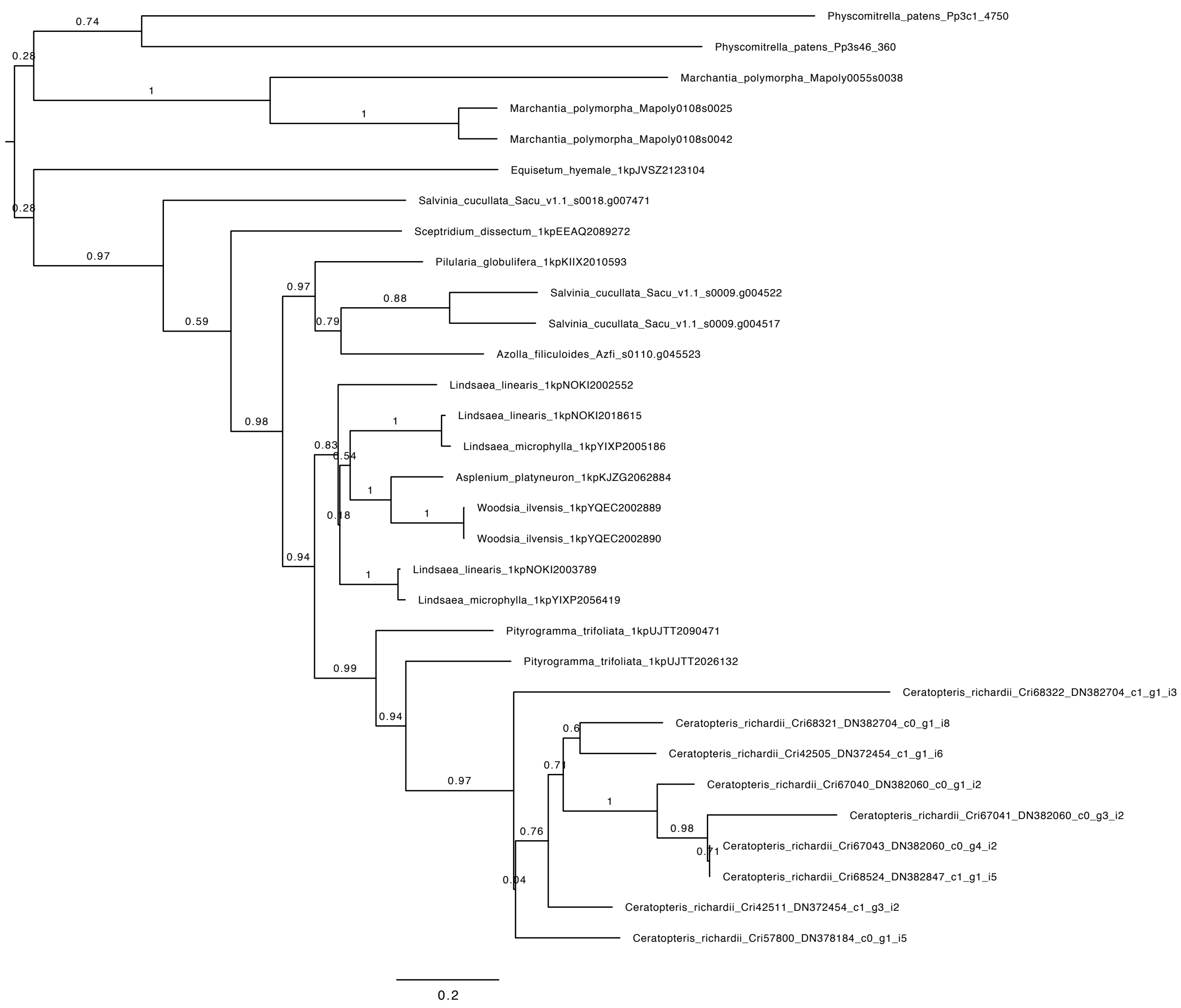

Fig S1. OrthoFinder FastTree phylogeny of OG0007870 containing hopanoid triterpene synthase homologs. Numbers above interior branches indicate the FastTree ultrafast bootstrap support for the proceeding node.

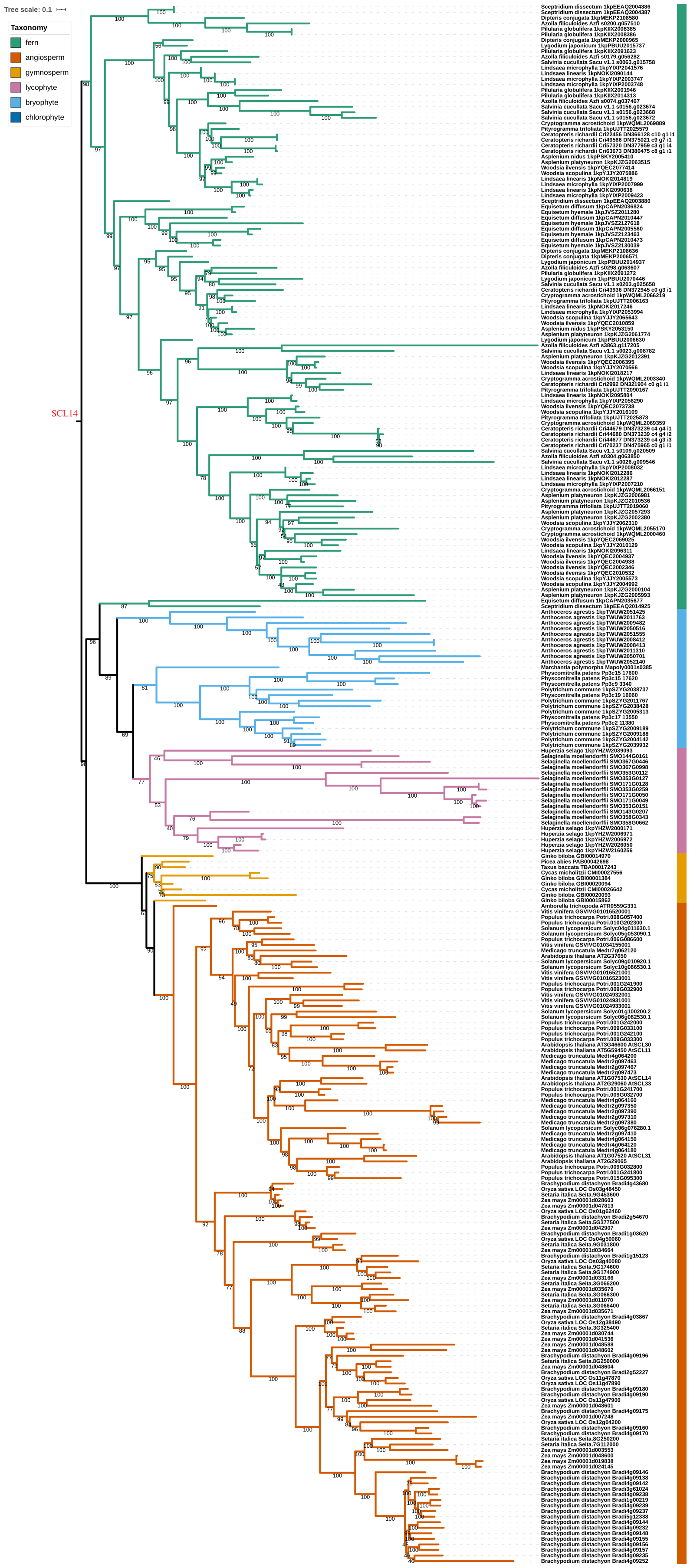

Fig S2. Phylogenetic analysis of orthogroups containing GRAS homologs. in OG0000320 (SCL14). The maximum likelihood tree was midpoint rooted, and numbers below interior branches indicate the IQ-TREE ultrafast bootstrap support for the proceeding node. The

branches and color bars (right) are color coded to reflect the taxonomic classification of each sequence.

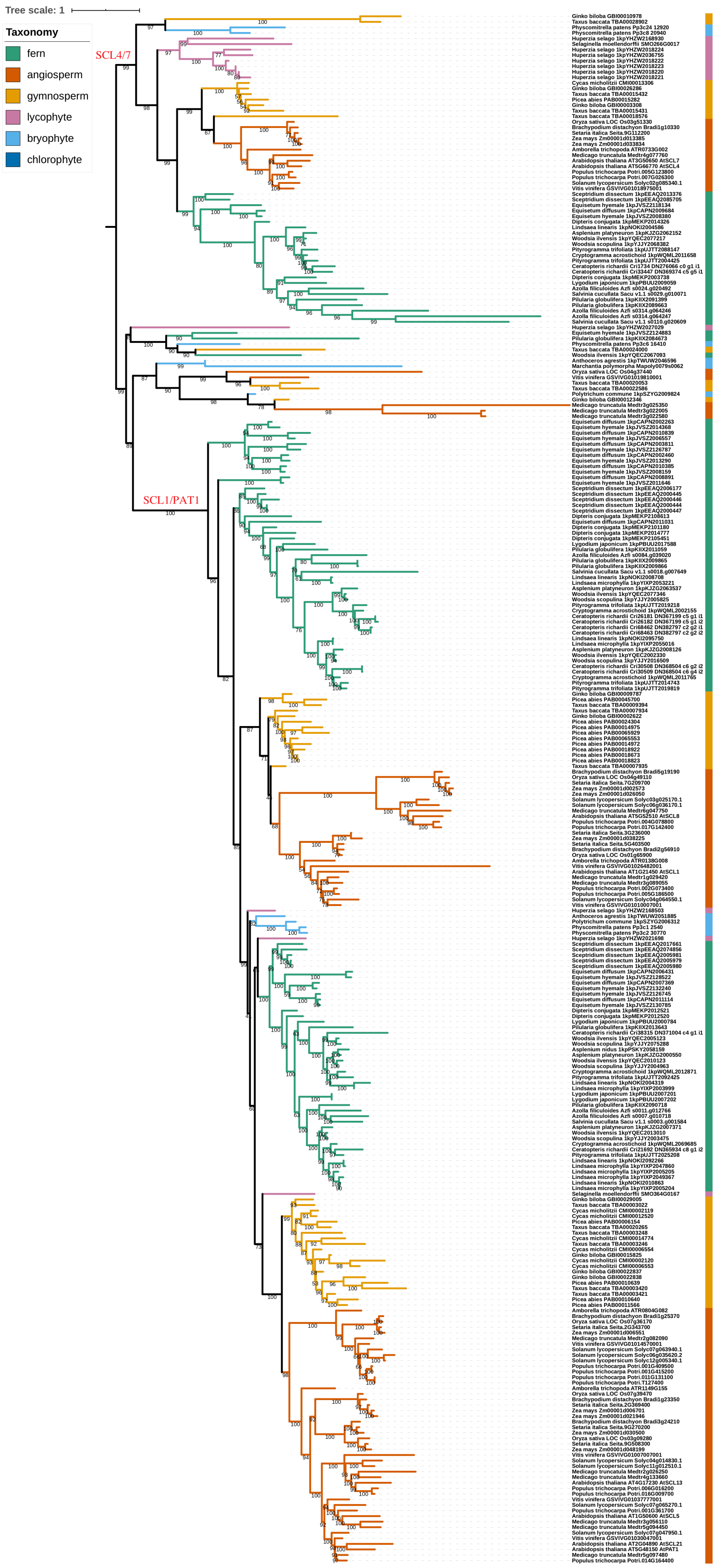

Fig S3. Phylogenetic analysis of GRAS homologs in OG0000323. Tree is displayed as in Fig S2.

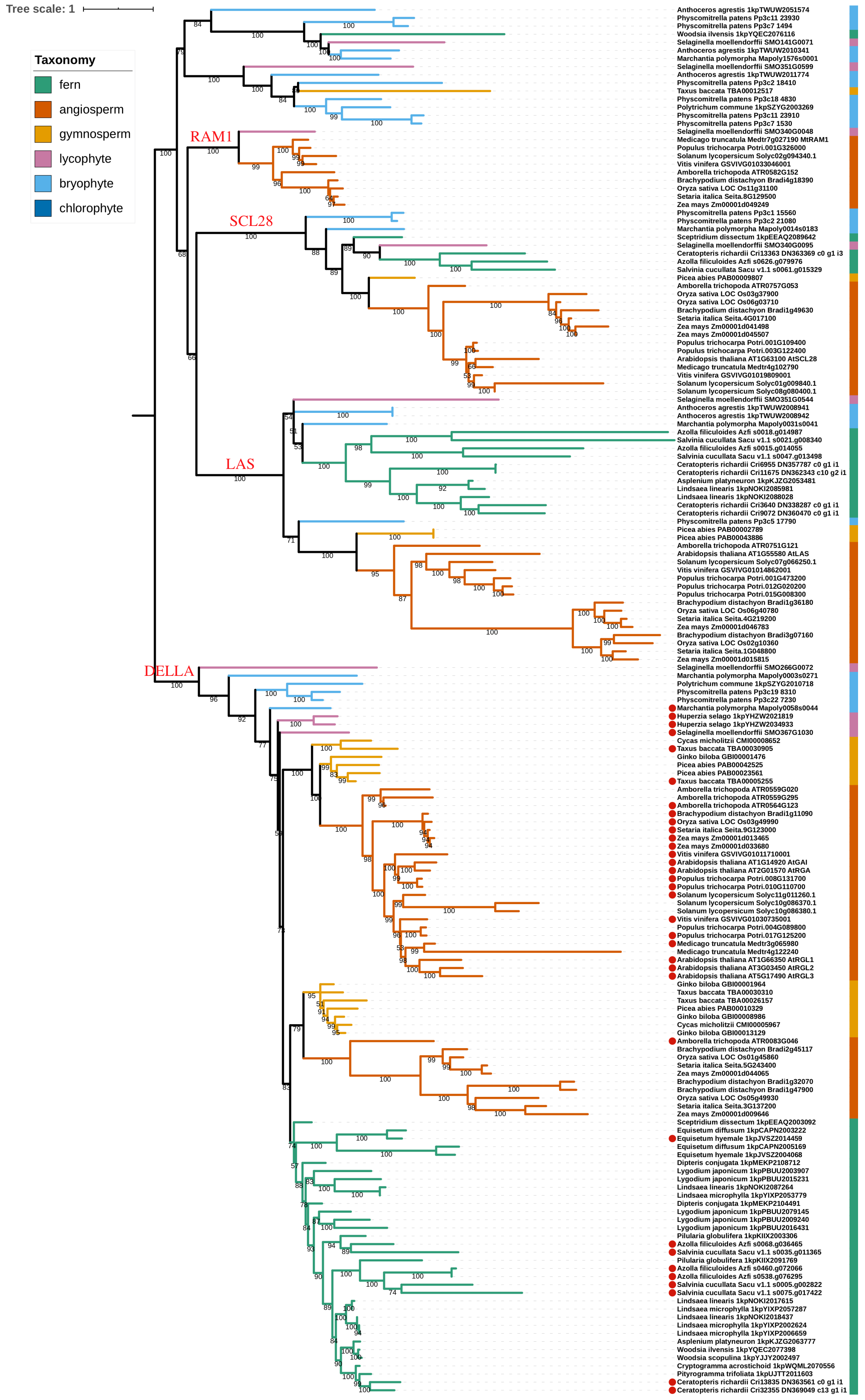

Fig S4. Phylogenetic analysis of GRAS homologs in OG0000755. Tree is displayed as in Fig S2. Red solid circles represent sequences with the DELLA domain (Pfam PF12041).

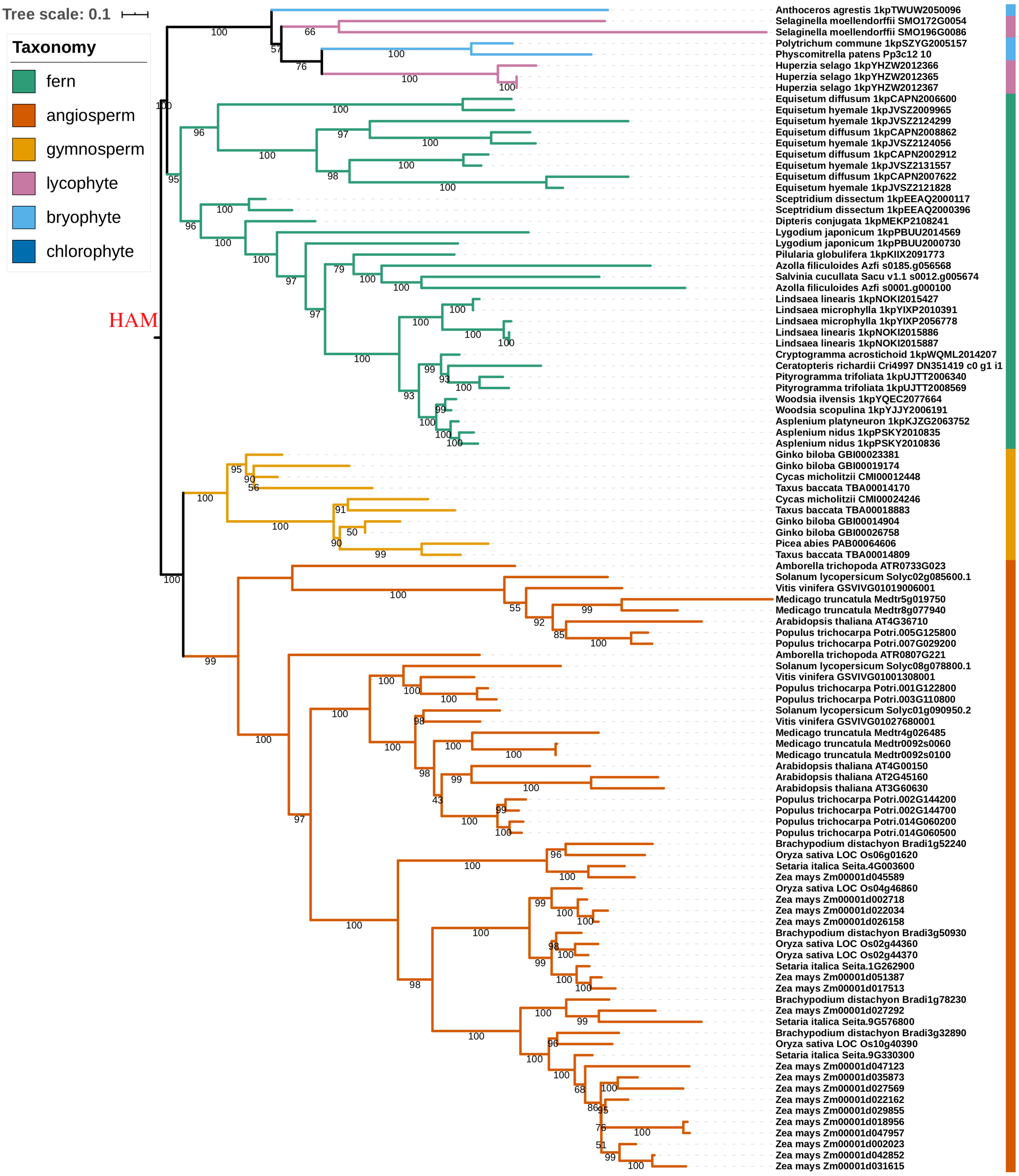

Fig S5. Phylogenetic analysis of GRAS homologs in OG0001609 (HAM). Tree is displayed as in Fig S2.

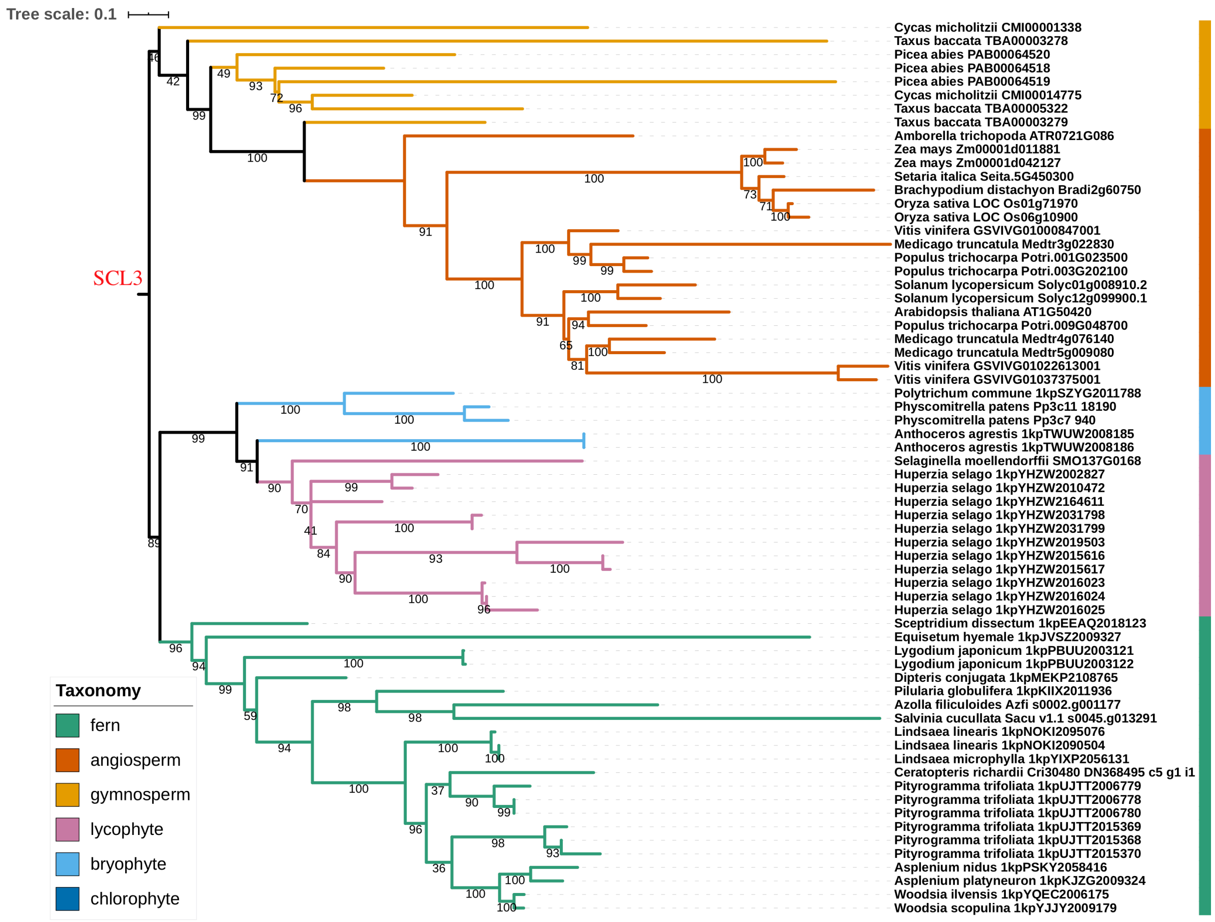

Fig S6. Phylogenetic analysis of GRAS homologs in OG0003235 (SCL3). Tree is displayed as in Fig S2.

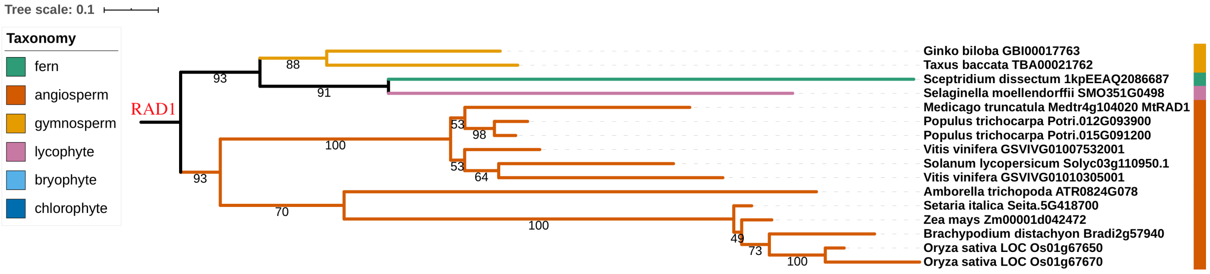

Fig S7. Phylogenetic analysis of GRAS homologs in OG0009315 (RAD1). Tree is displayed as in Fig S2.

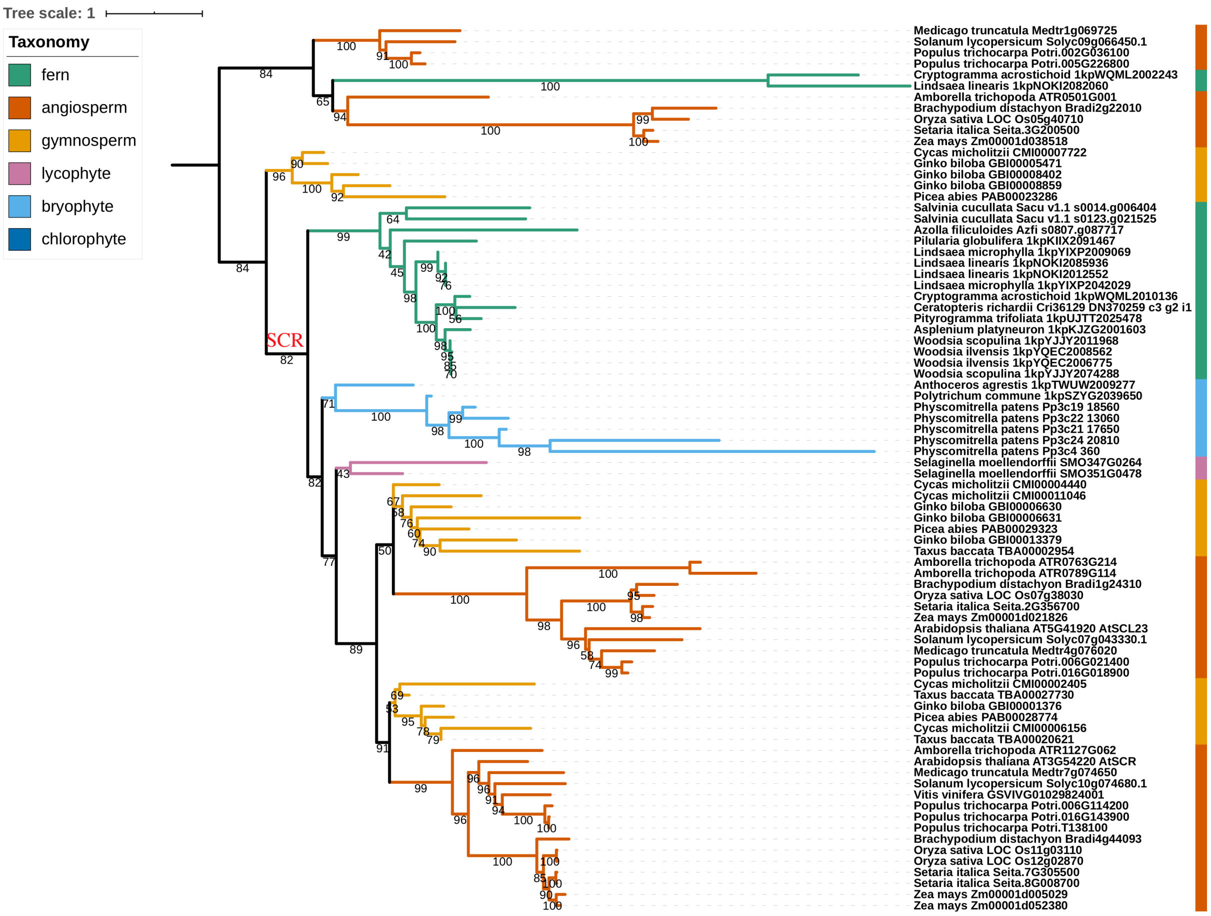

Fig S8. Phylogenetic analysis of GRAS homologs in OG0002549. Tree is displayed as in Fig S2.

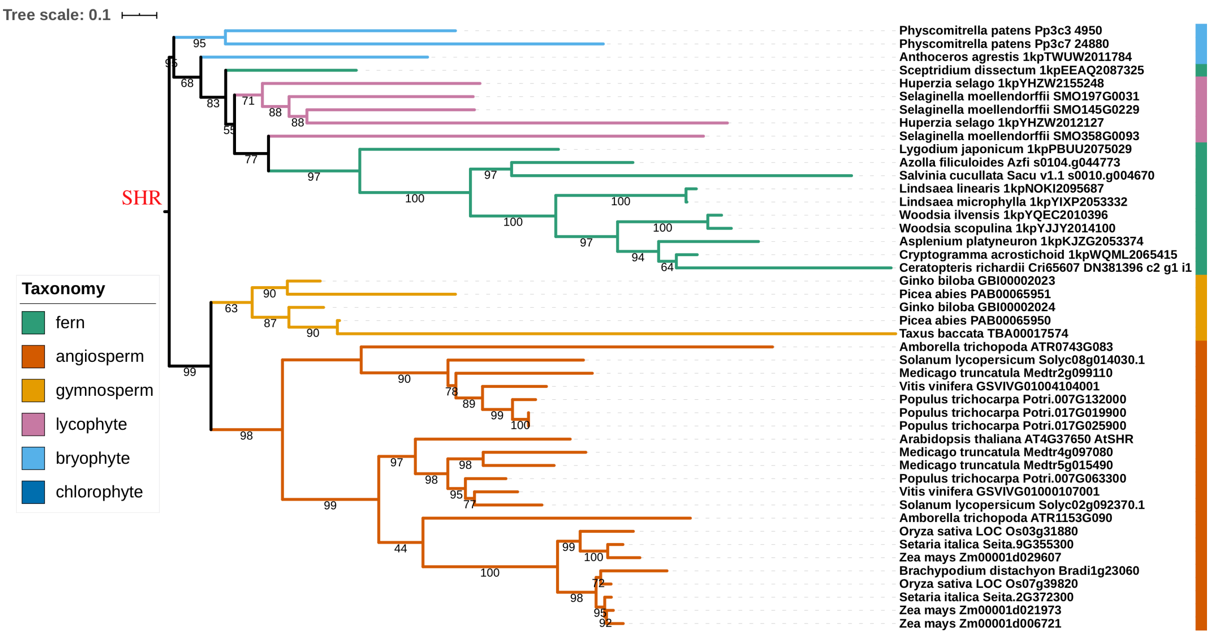

Fig S9. Phylogenetic analysis of GRAS homologs in OG0005632 (SHR). Tree is displayed as in Fig S2.

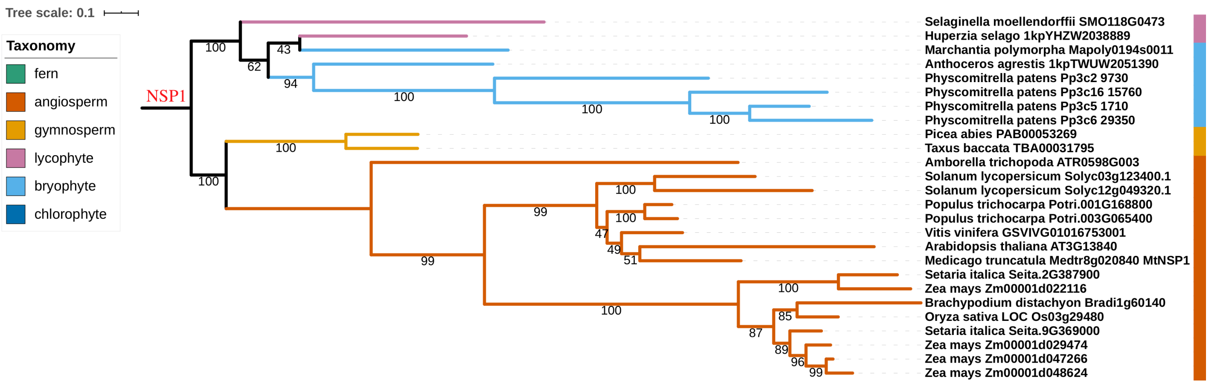

Fig S10. Phylogenetic analysis of GRAS homologs in OG0007977 (NSP1). Tree is displayed as in Fig S2.

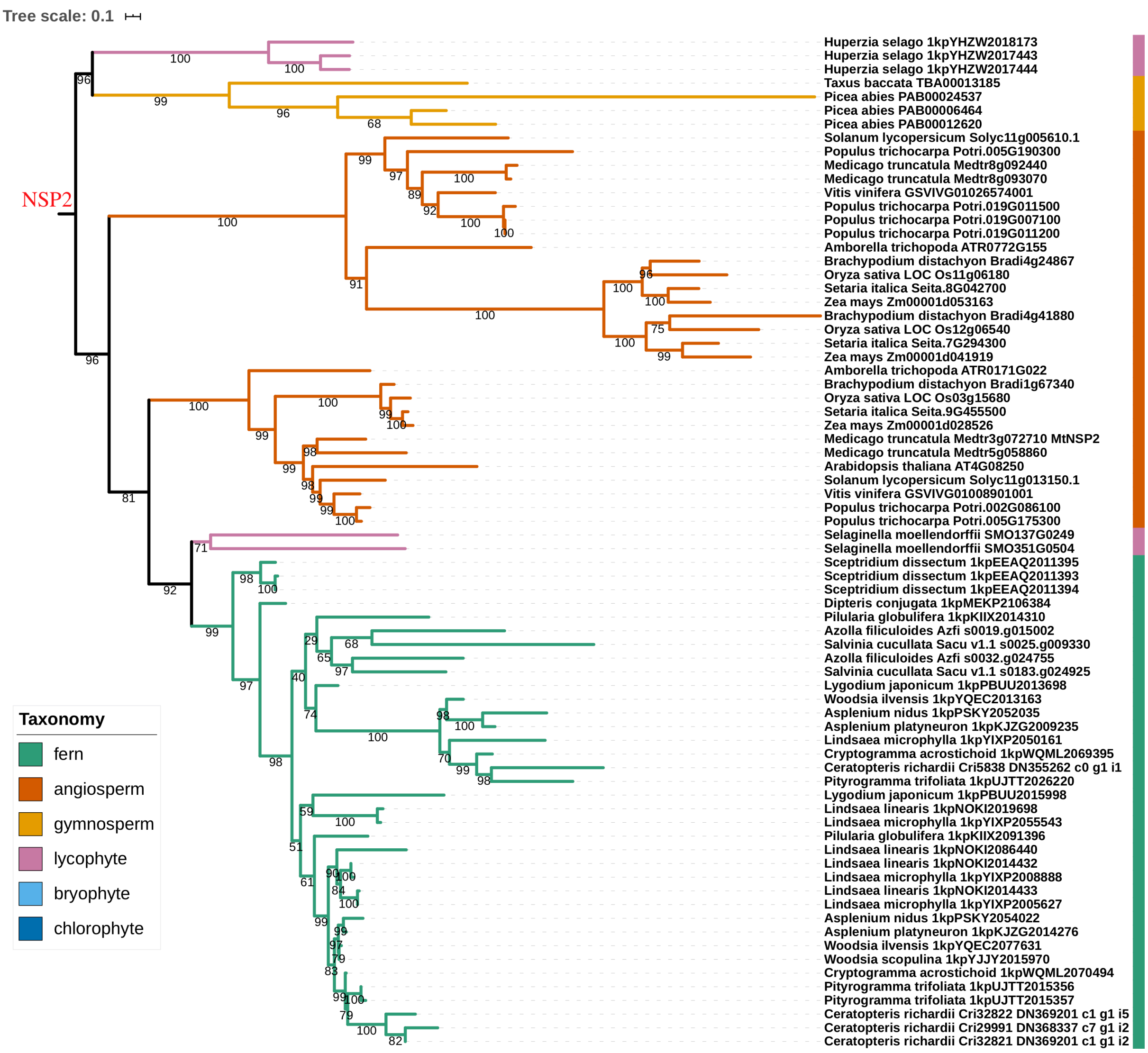

Fig S11. Phylogenetic analysis of GRAS homologs in OG0002843 (NSP2). Tree is displayed as in Fig S2.

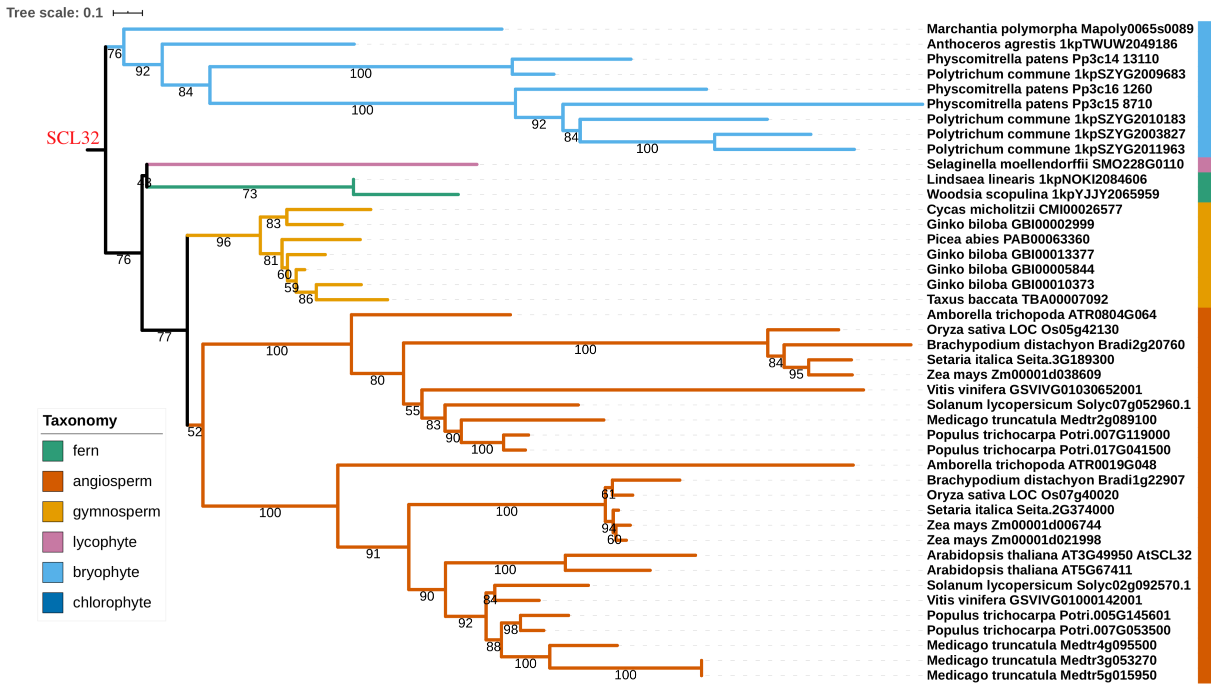

Fig S12. Phylogenetic analysis of GRAS homologs in OG0006078 (SCL32). Tree is displayed as in Fig S2.

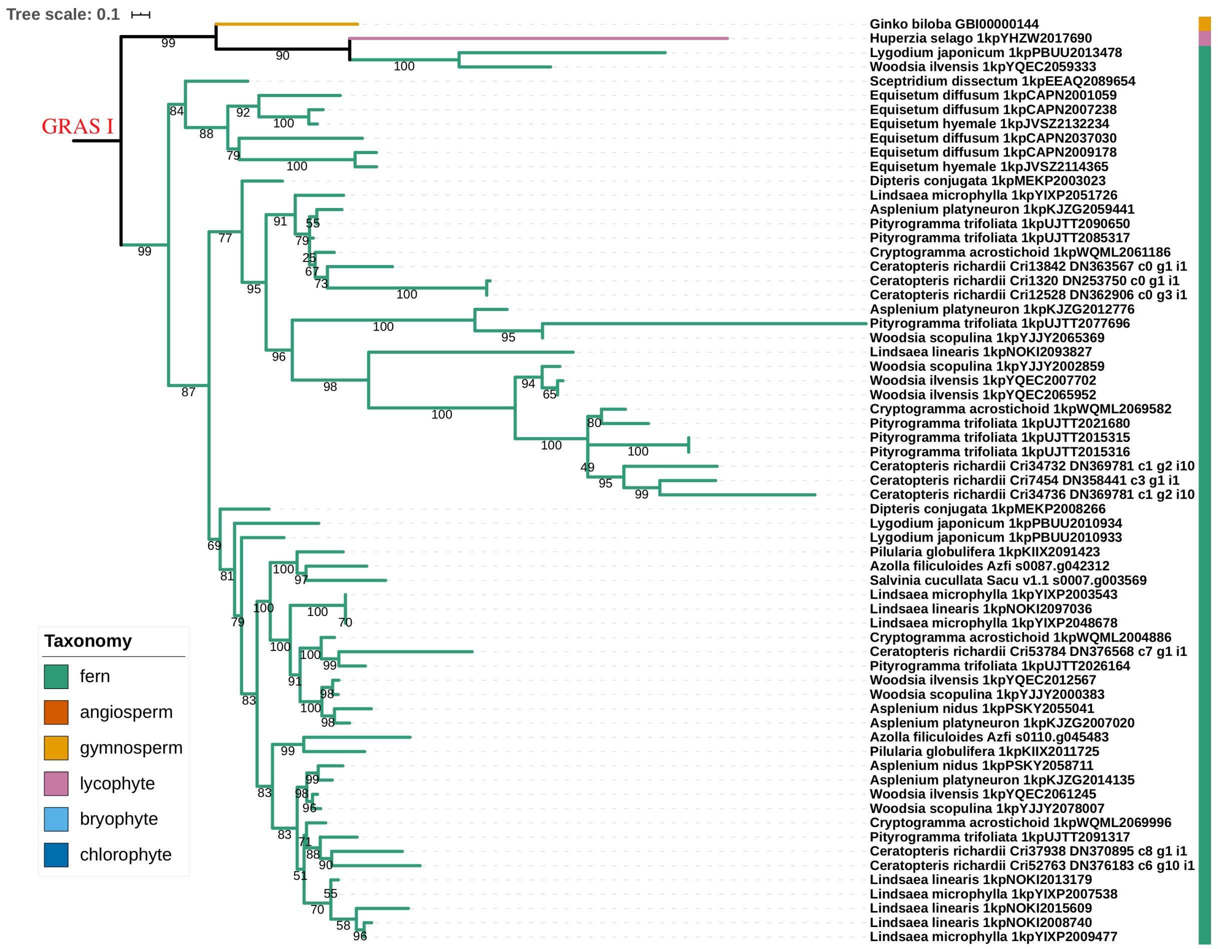

Fig S13. Phylogenetic analysis of GRAS homologs in OG0003479 (GRAS I). Tree is displayed as in Fig S2.

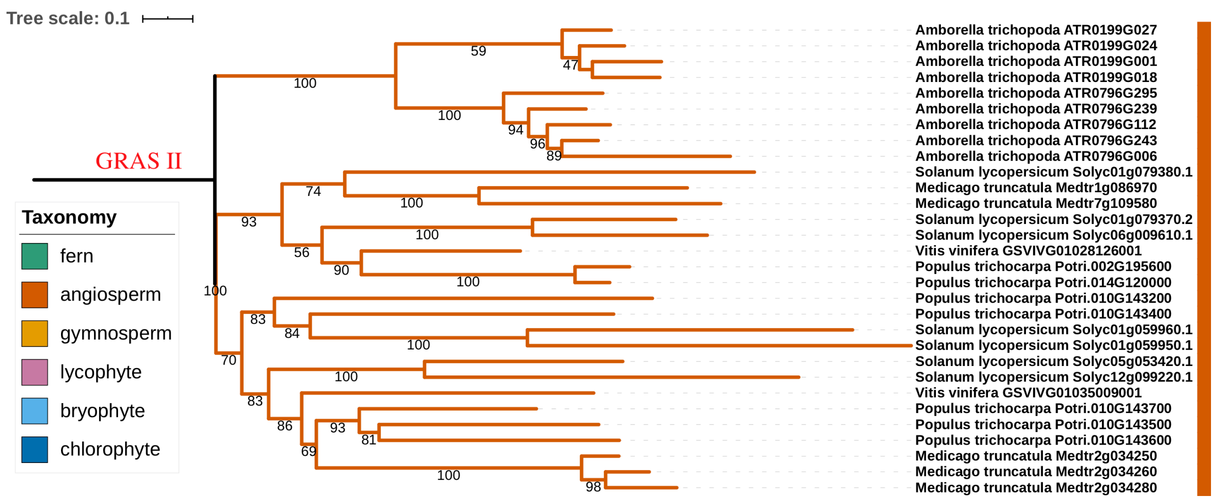

Fig S14. Phylogenetic analysis of GRAS homologs in OG0007617 (GRAS II). Tree is displayed as in Fig S2.

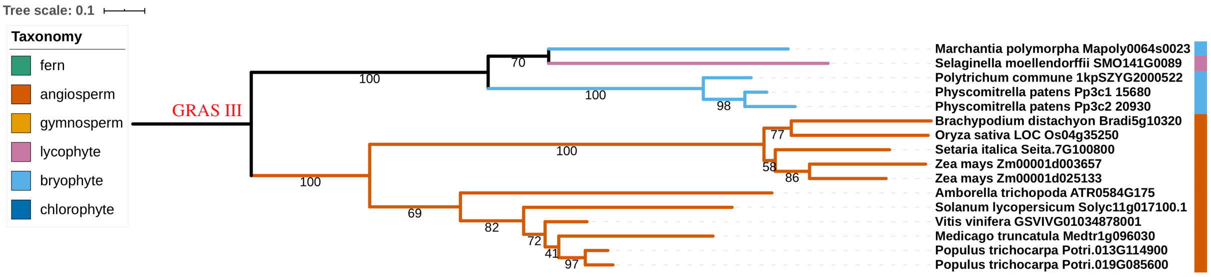

Fig S15. Phylogenetic analysis of GRAS homologs in OG0009292 (GRAS III). Tree is displayed as in Fig S2.

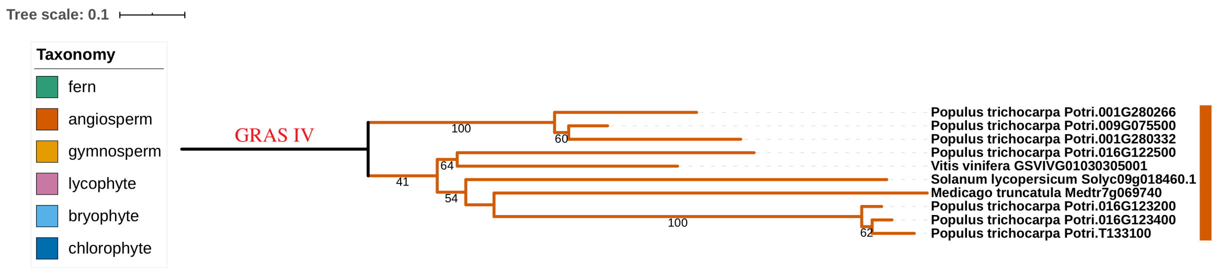

Fig S16. Phylogenetic analysis of GRAS homologs in OG0010265 (GRAS IV). Tree is displayed as in Fig S2.

Table S1. Summary of RNA sequencing data from different *Ceratopteris richardii* tissues.

Table S2. OrthoFinder orthogroup counts per species.

Table S3. Gene family evolutionary dynamics predicted using Dollo parsimony, Wagner parsimony, and OrthoFinder inferred gene tree duplications.

Table S4. Gene Ontology enrichment analyses

Note S1. OrthoFinder predicted gene families.

Notes S2-S16. Alignments of land plant GRAS proteins.
